# Students being and becoming scientists: measured success in a novel science education partnership

**DOI:** 10.1057/palcomms.2016.5

**Published:** 2016-03-01

**Authors:** Joanna Yang, Thomas J LaBounty, Stephen C Ekker, Chris Pierret

**Affiliations:** 1 Clinical and Translational Science, Mayo Clinic, Rochester, MN, USA; 2 LaBounty Consulting, LLC, Woodbury, MN, USA; 3 Biochemistry and Molecular Biology, Mayo Clinic, Rochester, MN, USA

## Abstract

The primary and secondary learning years shape development of scientific interest and skills required for science literacy, presenting a critical timeline target for science education intervention. Although many initiatives exist to target this timeframe, the modern classroom belies easy scientific investigation. Numerous initiatives often run simultaneously in a given classroom, creating limited capacity for variable control. Consequently, there is a dearth of high-quality and meaningful data in education sciences that exacerbates the general segregation of education research from practice. Many science reform programmes go unmeasured. The limited number that is researched often report strictly qualitative results or stop short of statistically significant quantitative investigation. Lack of high-resolution data restricts the ability to make informed policy changes and precludes attainment of “evidence-based education”. Here, we demonstrate 5-year efficacy of a novel, inquiry-based primary and secondary science reform programme Integrated Science Education Outreach (InSciEd Out). Five years of data over three cohorts of matched students from US grades 5–8 show maintained gains in science fair and honours biology election, as well as improved performance on Minnesota state standardized science testing. Detailed value-added analyses further reveal InSciEd Out-correlated gains in partnership-focused areas of life sciences, and history and nature of science. These analyses provide evidence that scientifically rigorous evaluation demonstrating relevant programme efficacy is indeed achievable in education science. Our results support the premise that the InSciEd Out programme is a scalable intervention capable of primary and secondary science education reform. The programme substantively builds upon prior efforts in the field. Although InSciEd Out deploys novel approaches and tools, the broad lessons learned from this programme are readily translatable to other contemporary efforts cultivating science literacy for all.

## Introduction

Science, technology and innovation (STI) drive progress in sectors from public health to security. Globally, STI is imperative to achieving the Millennium Development Goals (United Nations Economic and Social Council, 2013; United Nations, 2014); nationally, the United States (US) views STI as key to securing the nation’s future (National Science and Technology Policy, Organization, and Priorities Act of 1976). Although STI has contributed to > 50% of US economic growth post-World War II (Sturko Grossman, 2008), Science, Technology, Engineering and Mathematics (STEM) occupations account for only 5.5% of the US workforce (Langdon *et al.,* 2011). There is therefore much interest in bolstering the STEM pipeline by cultivating scientific interest during the primary and secondary years (Murphy and Beggs, 2003; Osborne *et al.,* 2003; Tai *et al.,* 2006; Osborne, 2007; Logan and Skamp, 2008).

Many initiatives exist to target the primary and secondary pipeline in schools. In response, the science education field has begun emphasizing rigorous measurement of student outcomes and greater consideration of study designs (Schroeder *et al.,* 2007; Slavin *et al.,* 2012). This push for rigour is welcomed, as major challenges in science education include both high-resolution capture of student outcomes (Schroeder *et al.,* 2007; Slavin *et al.,* 2012) and application of research data to practice (Porter and McMaken, 2009; Rust, 2009). One 1998 report found that less than 10% of teacher professional development programmes directly measured student achievement (Killion, 1998); another same-year math and science review found only four science programmes with data collection on student learning (Kennedy, 1998). More recent summations of the field show how lack of scientific rigour remains a barrier to evidence-based practice. Although calls for scientific teaching are widespread, actual practice of scientifically rigorous evaluation of science education is largely lacking (Handelsman *et al.,* 2004; Hanauer *et al.,* 2006; Wieman, 2007).

Part of the difficulty of capturing student gains lies within the myriad confounders embedded within the modern classroom. This challenge can often result in reduced statistical power due to limited sample size. It can also lead to reductions in the scope or depth of the tested intervention. One example is the concurrent implementation of multiple different programmes in every classroom as part of school or District-wide initiatives. This practice is a practical reality, but it presents the perfect catch-22: too many initiatives obstruct progress (Hatch, 2002; Fullan, 2004; Bartalo, 2012; DuFour *et al.,* 2013; Freedman and Cecco, 2013; Smith, 2015), but simultaneous initiatives complicate selection of effective programming. Identifying a signal in this noise requires detailed quantification of value-add in a manner that both celebrates and accounts for the everyday classroom. There is therefore a need for effective statistical evaluation in education science to test correlations in student outcomes with specific programming. Selection of successful, evidence-based programmes is needed to better build the primary and secondary science pipeline for the future.

Herein we evaluate a school-wide, inquiry-based science education intervention Integrated Science Education Outreach (InSciEd Out, insciedout.org). InSciEd Out is a collaborative partnership committed to rebuilding primary and secondary science education curricula for the twenty-first century. The programme is driven by scientific professional development internships for multidisciplinary teams of primary or secondary teachers from a common school. Internships are followed by sustained support in curriculum writing and implementation during the school year (Pierret *et al.,* 2012). Teacher professional development remains a widely accepted method to strengthen the STEM pipeline (Kennedy, 1998; Guyton and Dangel, 2004; Czerniak *et al.,* 2006; Schroeder *et al.,* 2007; Lumpe *et al.,* 2012; Shymansky *et al.,* 2012; Slavin *et al.,* 2012), but numerous aspects distinguish InSciEd Out from other professional development offerings.

First, InSciEd Out is a sustained partnership. The programme recruits whole teaching teams, not just science teachers, to cultivate a school culture of change. It then fosters connections between participant schools and their larger communities. The presence of school-to-community connections has been shown to correlate with improved student learning and behaviour (Michael *et al.,* 2007). InSciEd Out’s status as a partnership between schools, scientists, university faculty and parents follows recommendations to sustain professional development in science education (National Science Teachers Association, 2006). The long-term nature of InSciEd Out professional development and its ongoing support infrastructure are also designed to rectify key pitfalls of ineffective professional development programming (Gulamhussein, 2013).

Second, one signature component of InSciEd Out is the extensive use of the aquatic animal the zebrafish (*Danio rerio*). Teacher interns spend considerable laboratory time exploring science through the zebrafish model system. In turn, the curricula they create incorporate zebrafish for student exploration of science. Zebrafish have been previously used effectively in inquiry-based classroom activities (Ekker, 2009). The model system is highly adaptable to the school environment due to its high fecundity, transparent external embryonic development, genetic similarities to humans, size, availability of tissue-specific transgenics and timeline of development (Kimmel *et al.,* 1995). Of the above characteristics, transparent development has been shown to be especially effective in challenging students’ Life Sciences misunderstandings, particularly with regard to cells and heredity (Berthelsen, 1999).

Access to model systems like the zebrafish lends to InSciEd Out’s status as a unique platform for inquiry-based science. Although inquiry-based learning is commonly cited in both professional development and education reform (Keys and Bryan, 2001; Anderson, 2002; Capps *et al.,* 2012; Furtak *et al.,* 2012), InSciEd Out’s implementation of learner-driven inquiry is distinguishable in both scale and depth. InSciEd Out strives to realize the version of inquiry represented by Short (2009) as “a collaborative process of connecting to and reaching beyond current understandings [...]”. InSciEd Out additionally believes that inquiry begins with a question or point of perplexity that is intriguing to a learner and involves a complex journey towards deeper understanding. Science is the application of a structure to ask and answer a question through inquiry. Inquiry is therefore essential to science education because you can teach science to learners, but without inquiry learners cannot be scientists. The fundamental expectation of InSciEd Out is that learners should be producers of novel science knowledge. InSciEd Out learners conduct peer-reviewed and publishable research, where the scientific outcome is truly unknown. To this end, both teacher interns and their primary and secondary students are supported to ask and answer their own new questions in science. This expectation of learners to strive for personal and novel science pushes learners towards self-direction on the National Academy of Science’s Essential Features of Classroom Inquiry spectrum (Olson and Loucks-Horsley, 2000).

Ultimately, InSciEd Out is driven by a detailed theory of action. The above foci upon interdisciplinary partnership and student-driven inquiry are but two cornerstones of InSciEd Out’s detailed theory of action. Many modern science education reform efforts are driven by incomplete theories of action (Fullan, 2006). InSciEd Out instead strives to explicitly state, understand and assess the strategies it employs. Many different theories and strategies shape InSciEd Out, and the programme is continuously revising itself alongside best practice. Nevertheless, InSciEd Out strives to follow the seven principles set forth by Michael Fullan in pursuit of meritorious change: (1) A focus on motivation; (2) Capacity building; (3) Learning in context; (4) Changing context; (5) A bias for reflective action; (6) Tri-level engagement; (7) Persistence and flexibility (Fullan, 2006).

InSciEd Out teacher professional development is structured as sequential tiers of internships. Tier 1 internships are 12 days of instruction and exploration in a thematic area, followed by an additional 3 days of curriculum development. Tier 1 teacher interns learn about community-generated health themes, genetics and development, pedagogy, dialogue and the nature of science. Tier 2 internships enable an additional 5 days of independent integration of cultural relevance into the initial scientific work developed during Tier 1. One key goal of Tier 2 learning is to revise InSciEd Out classroom curricula to reach students previously marginalized to STEM disciplines. Lastly, the Tier 3 Gold Master internship is an intensive, opt-in programme for teachers who wish to become InSciEd Out Teacher Leaders. Training spans the course of 2 years and is focused around capacity building in inquiry, action research, collaborative peer review and global awareness.

InSciEd Out curricula created within the internships range from a few lessons to a months-long experience integrated among disciplines such as Language Arts, Mathematics, Science, Physical Education and Art. The lessons are driven by state standards and are cross-matched to Next Generation Standards. Each unique set of InSciEd Out curriculum is called a module and is designed by teacher interns in partnership with InSciEd Out team members. Modules replace previously inefficient or outdated lesson plans. In this manner, high-quality student learning experiences are made possible without overtaxing content-saturated syllabi. An excerpt from the rubric for a module is included in Table 1.

The study here analyses outcomes for InSciEd Out partner school Lincoln K-8, Rochester, MN. Lincoln is part of the Rochester School District (MN#535) and has been an InSciEd Out partner since 2009. A previous preliminary analysis of 2-year InSciEd Out implementation at Lincoln revealed improvements in Lincoln student performance on the Minnesota Comprehensive Assessment (MCA) Science relative to the state and other District schools. Longitudinal analysis from 2008 to 2011 revealed increases in student science proficiency. Effect size analysis normed to the state of Minnesota showed that the first cohort of InSciEd Out Lincoln students outpaced District students on MCA Science improvement from grades 5 to 8. Multiple linear regression analyses controlling for demographics further showed that the level of Lincoln student MCA Science growth exceeded that of other schools’ students in the District. InSciEd Out students at Lincoln also showed improvements in their science engagement through simultaneous increases in honours biology election and science fair participation (Pierret et *al.,* 2012). While these results were promising and trended towards gains in student science learning, statistical significance was not achieved in these analyses.

This current analysis is a longitudinal, multi-cohort study of InSciEd Out’s quantitative value-add at Lincoln, spanning 5 years of programme implementation. Previous analysis (Pierret et *al.,* 2012) focused upon the 2011 grade 8 cohort (Cohort 1). Here, we analyse the 2012–2014 grade 8 cohorts (Cohorts 2–4), utilizing Lincoln students as their own internal controls and the broader Rochester Public Schools District (MN#535) and the state of Minnesota as externally normed comparisons. This study expands upon the previous pilot analysis of Lincoln to comprehensively evaluate InSciEd Out as a programme for science education achievement. In addition, the broader significance of these results is presented to emphasize the attainability of and need for appropriate and detailed statistical methods. These methods aid in capture of statistical significance for science education programming.

## Methods

### Engagement metrics

The first level of analysis involves engagement data including overall eligible Lincoln student cohorts pre- and post-InSciEd Out to depict overall trends. The percent election of honours biology and percent participation in regional science fair are calculated as the proportion of eligible Lincoln students engaging in the science pipeline. All enrolled grade 6–8 students are included for science fair analysis, and graduating grade 8 students are included in the honours biology analysis. Science fair allows students to voice ownership of their science. Honours biology election is an important self-selected science class decision that historically determined downstream high school science trajectory in the Rochester Public School District.

### Achievement metrics

Longitudinal cohort achievement analysis utilizes individual- level data for Lincoln students’ performances on grade 5 versus grade 8 MCA tests for each cohort. Demographics for the Lincoln student cohort were drawn from grade 5 school records. Publically available summary data for the state of Minnesota, District and individual District schools were obtained from the Minnesota Department of Education (accessible at: http://education.state.mn.us/MDE/Data/). Grades 5 and 8 time points were chosen due to administration of MCA Science in grades 5,8 and high school. At the time of our study, high school MCA proficiency in mathematics and reading were two requirements of graduation. RPS has no minimal graduation requirement for science, much less a high-stakes middle school equivalent; the MCA Science remains the only Minnesota standards-based accountability assessment. Recent legislative changes post-study have since relaxed mathematics, writing and reading graduation requirements to first phase out use of the MCA test in favour of the ACT and then to eliminate mandatory graduation assessments entirely (Minnesota Department of Education, 2015).

#### Overall assessment

Analysis of overall MCA performance first compares grades 5 and 8 cohorts directly without matching students via percent proficiency. The MCA has four achievement levels: Exceeds Standards (E), Meets Standards (M), Partially Meets Standards (P) and Does Not Meet Standard (D). Percent proficient is the percentage of students at level M or E.

#### Strand analysis

Subsequent strand analysis utilizes *z*-scores, which are standard, state-centered scores representing the number of standard deviations any given data point is above or below the mean. While z-scores cannot wholly compensate for construct differences and use of normative growth does not reflect absolute growth in science knowledge, normative growth is common in programme evaluation. *Z*-scores enable analysis precluded by grades 5 versus 8 standards differences, versioning of the MCA test (II versus III) and raw versus stanine reporting of strand scores for different years. Strand analysis is matched, as some students enrolled in grade 5 may not remain enrolled in grade 8. Student ID individually matches students from grade 5 to grade 8 with October grade 8 enrolment providing school affiliations. Matched strand analysis uses *z*-score data from Lincoln school records for individual matching of students from grades 5–8 and only includes students continuously enrolled at Lincoln for the study timeframe. Data for other middle schools and the District (MN#535) are from District records and are also individually matched.

#### Multiple linear regression

A series of multiple linear regressions examine the value-added contribution of Lincoln enrolment during InSciEd Out programme implementation. These models control for grade 5 MCA Science scores, demographics (gender, ethnicity, limited English proficiency, Special Education status, and Free or Reduced Price Lunch) and Lincoln enrolment to predict grade 8 MCA science scores. Each regression model is completed twice. The first is a “null” model with only the grade 5 score and other demographic covariates included in the fit. The second, “full” model includes a dichotomous variable indicating enrolment at Lincoln. As Lincoln enrolment during this period coincides with InSciEd Out implementation, it serves as a surrogate marker for InSciEd Out effect. Regression estimates effects of being enrolled at Lincoln with *R*^2^ values calculating explained variance for each model. The *F*-test of change, or the *F*-statistic of the ANOVA test, compares the explanatory powers of the null versus full regression models. This identifies whether or not the inclusion of additional explanatory variables to the null model results in a significant increase in explained variance. This metric therefore detects the statistical significance resulting from the inclusion of the Lincoln K-8 Choice indicator in our study. When comparing null and full regression models, the change in *R*^2^ quantifies the additional explained variance resulting from adding the additional explanatory variables to the null model. In this study, the *F*-statistic and change in *R*^2^ determine effects of being enrolled at Lincoln. For a more conservative estimate of statistical significance, Bonferroni correction for multiple comparisons is provided to adjust for the number of variables in each regression model. This new level of significance is *P* = 0.005 (original *P* = 0.05/9).

This research was reviewed under the Mayo Clinic Human Research Protection Program by the Mayo Clinic Institutional Review Board and deemed exempt. InSciEd Out’s use of zebrafish as a platform for student inquiry was approved by the Mayo Clinic Institutional Animal Care and Use Committee.

## Results and discussion

**Figure 1 |:**
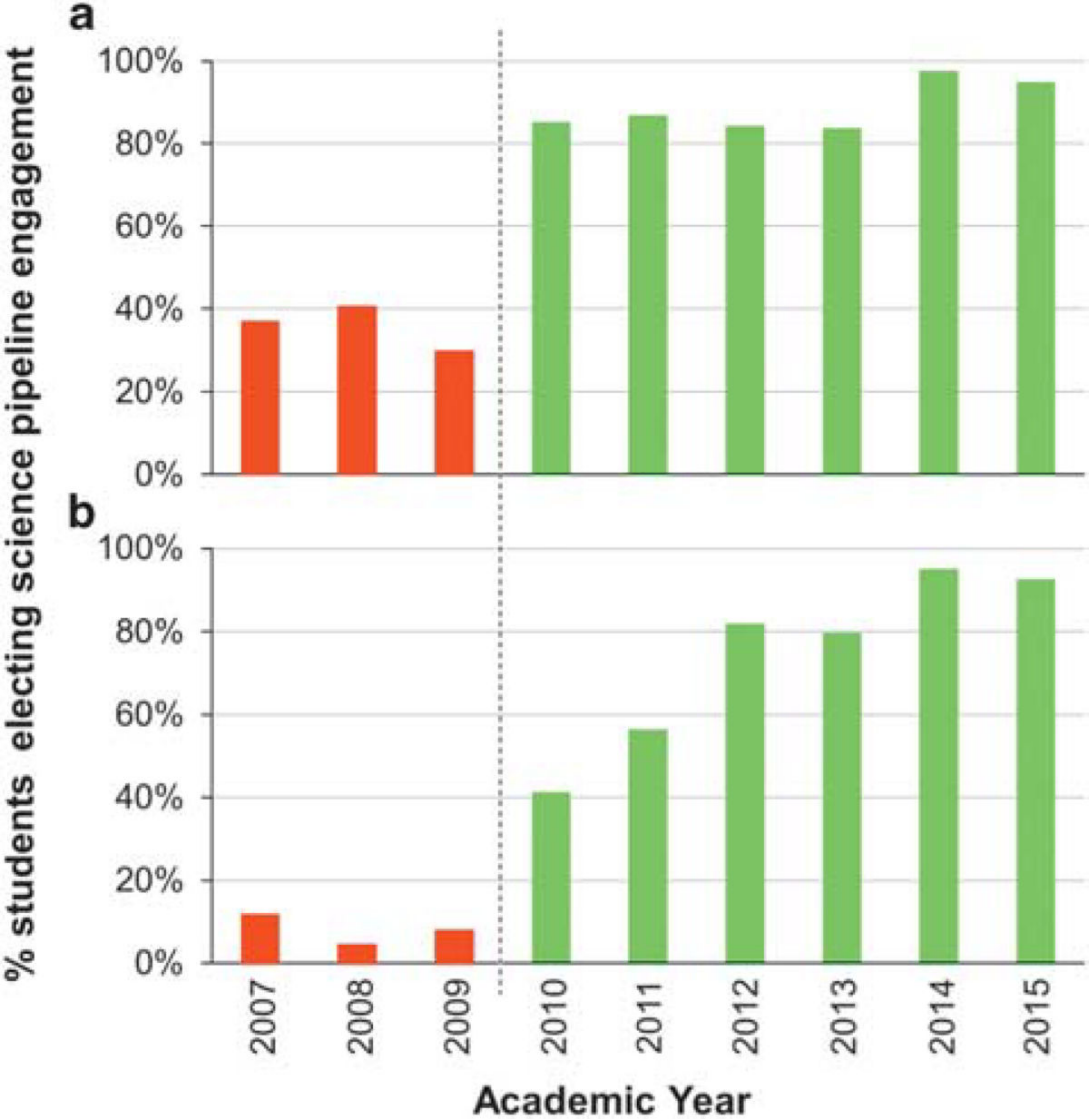
Percent of Lincoln students electing science pipeline engagement pre- and post-InSciEd Out implementation. (a) Student election of honours biology. Percent election calculated out of total number of eligible grade 8 students; (b) student participation in science fair. Percent election calculated out of total number of eligible grades 6–8 students. *Note*: Three years pre-data (red) and 6 years post-data (green) are portrayed. Raw numbers for both engagement metrics are provided in supplementary Tables S1 and S2.

Engagement analysis reveals sustained improvement of Lincoln students’ science pipeline election correlated with InSciEd Out. Figure 1 shows baseline data from the 2006–2007 school year through the 2008–2009 school year and post-InSciEd Out implementation data from the 2009–2010 to 2014–2015. Election of honours biology is at 95% in 2014–2015 (6 years post-initial InSciEd Out implementation), up from a baseline of 37% in 2007 (Fig. 1a). Science fair participation post-InSciEd Out implementation also shows continued improvement at 93% in 2014–2015, up from an initial 12% in 2007 (Fig. 1b). Statistical analyses comparing overall pre-post numbers for both metrics show significant increases post-InSciEd Out (*P*<0.001).

**Figure 2 |:**
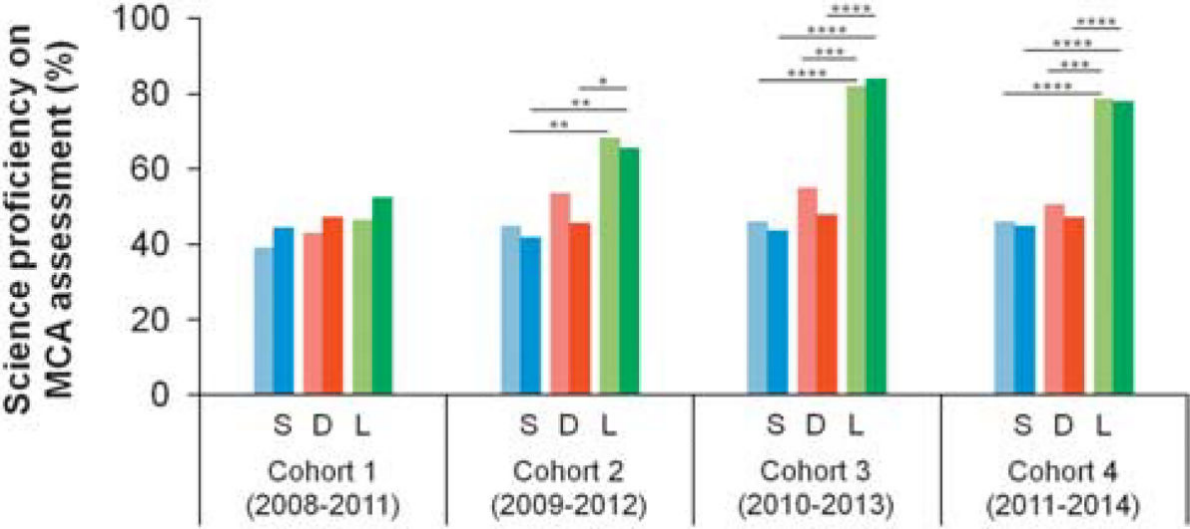
Longitudinal science learning proficiency comparison. *Note*: State (S), District (D) and Lincoln (L) grades 5 (lighter colour, left bar) and 8 (bolder colour, right bar) MCA Science test percent proficiencies are provided for four student cohorts. Years of InSciEd Out implementation are grades 7 and 8 for Cohort 1, grades 6–8 for Cohort 2, grades 5–8 for Cohort 3 and grades 4–8 for Cohort 4. Statistical analysis conducted via χ^2^ tests, *P<0.05, **P<0.01, ***P< 0.001, ****P≤ 0.0001.

The MCA Science test provides programme assessment insights extending engagement data to science learning. Overall analysis via the MCAs (Fig. 2) shows Lincoln emerging with statistical significance above the state and the District. InSciEd Out-correlated statistical significance emerges in Year 1 of implementation for grade 5 and in Year 3 for grade 8. Degree of significance is heightened and/or maintained with additional years of InSciEd Out programming. Despite these gains over time, comparisons of grade 5 versus grade 8 percent proficiencies are not statistically significant for any unique Lincoln cohort—posing a question as to where Lincoln’s “within-cohort” gains may be found.

Deeper analysis consequently accounts for the four content areas, called strands, within the MCA Science test: History and Nature of Science (HNS, MCA-II) or Nature of Science and Engineering (NSE, MCA-III), Physical Science (PSCS), Earth and Space Science (ESS) and Life Science (LIFS). InSciEd Out’s partnership with Lincoln in this study focused on HNS/NSE and LIFS to target historical performance issues. To better understand student outcomes attributable to the InSciEd Out intervention, in-depth strand analysis and multiple linear regression are conducted here, utilizing *z*-score conversion to standardize student scores to the state and allow for normative growth analysis. We evaluate data through two lenses: longitudinally, comparing each year to the last, and “within-cohort” by following unique groups of Lincoln students as they advance from grade 5 to grade 8. This “within-cohort” lens includes previously unpublished student data from 2009–2012 (Cohort 2), 2010–2013 (Cohort 3) and 2011–2014 (Cohort 4). Cohort 1 (2008–2011) was previously described by Pierret et *al.* (2012).

Student individually matched strand analysis (Table 2) reveals targeted Lincoln gains in InSciEd Out partnership strands of HNS/NSE and LIFS. Longitudinally, changes in Lincoln HNS/NSE strand scores show upward trends with increasing InSciEd Out exposure (−0.131, 0.101 and 0.197 Δ*z*-score chronologically). These numbers show Lincoln to be the only school in the District to improve its HNS/NSE relative standing to the state in Cohort 3. HNS/NSE improvement still exceeds that of the only other positive school in Cohort 4. LIFS *z*-scores show similar trends and are positive for all cohorts, indicating maintained gains (0.107, 0.391 and 0.200 Δ*z*-score chronologically). LIFS improvement exceeded that of other schools in Cohort 3 and is exceeded by only School 4 in Cohort 4. Raw *z*-score analysis shows that Lincoln still outscores School 4 in this year (supplementary Table S3, 0.735 and 0.730 *z*-score, respectively).

As Δ*z*-scores are intrinsically reflective of “within-cohort” progress, positive Δ*z*-scores at Lincoln are suggestive of Lincoln’s “within-cohort” gains (Table 2). Lincoln Cohorts 2 and 3 exhibit “within-cohort” gains in HNS/NSE; all Lincoln cohorts show these gains in LIFS. In the two strands not targeted by InSciEd Out programming, Lincoln showed ESS declines relative to the state in all cohorts and PSCS declines in Cohorts 2 and 3. Nevertheless, ESS and PSCS strand scores remain relatively high compared with both state and District scores (supplementary Table S3). These results show a specific HNS/NSE and LIFS effect strongly correlated to InSciEd Out programming.

Multiple linear regression extends targeted strand score gains to demonstrate statistical significance of Lincoln’s “within-cohort” student growth. Table 3 and supplementary Table S4 provide summary information from 40 regression models. These models include information for each of the three grade 5 to grade 8 cohorts and all cohorts combined, as well as both individual strand and overall modelling. *R*^*2*^ statistics show highest explained variance for the “All Strands” model with lower explained variance for individual strand modelling. This can be attributed to the low number of questions used to assess each strand, which impedes reliability of the strand data. Analysis of regression coefficients (*β*) reveals that Cohort 2 exhibits positive, but not statistically significant, growth attributable to Lincoln enrolment in HNS/NSE (0.150, *P =* 0.268) and LIFS (0.196, *P =* 0.198). Cohort 3 students have similarly positive, but non-significant HNS/NSE growth (0.217 *z*-score, P = 0.142), but statistically significant LIFS growth (0.452 *z*-score, *P* = 0.002). Cohort 4 students statistically significantly outscore predicted values in both HNS/NSE (0.375, P = 0.002) and LIFS (0.481 *z*-score, *P* = 0.000). Together, these results corroborate the strand analysis. There are both longitudinal (increasing statistical significance over time) and “within-cohort” (positive *β*) improvements in statistical significance of the Lincoln enrolment predictor. All-strand modelling shows Lincoln students scoring about where they would be predicted to score for Cohorts 2 and 3 (−0.040 and − 0.010 *z*-score) and nearly higher than predicted for Cohort 4 (0.173 *z*-score, *P* = 0.06). Thus, this growth is again specific to the targeted HNS/NSE and LIFS strands.

The *F*-test of change and *R*^2^ change statistics demonstrate corresponding statistical effects (*P* of Δ*R*^2^) in Cohort 3 LIFS (*P* = 0.002) and Cohort 4 HNS/NSE (*P* = 0.002) and LIFS (*P* = 0.000). After applying Bonferroni adjustment based on number of variables in each regression model (*P* = 0.005), results are still significant for Lincoln. Longitudinal trace substantiates previous analyses with increasing magnitude and significance for the *F*-test of change in HNS/NSE and LIFS over time.

## Conclusion

Overall, these data strongly support the premise that InSciEd Out is an efficacious science education intervention for Lincoln. Students maintain high status in state science assessments and engagement metrics with growth in InSciEd Out-targeted areas. As InSciEd Out activities in HNS/NSE and LIFS were designed within existing curriculum, they did not take away curricular focus upon ESS and PSCS. Thus, they cannot directly account for any noted declines. Future InSciEd Out partnerships will expand fields of science education focus and has begun with the 2014–2015 launch of an Environmental Sciences module. InSciEd Out expansion is ongoing in the District, Minnesota, broader US, India and beyond.

Given the dynamic modern-day classroom, signal isolation from the noise is difficult, but necessary, for science education advancement. This study reveals two important points to help select successful reform initiatives. First, “best practice” study design varies with study intent. Our pre-post assessment of multiple Lincoln cohorts in comparison with District and state enabled unbiased assessment of student performance despite not using conventional study design hierarchies (Coalition for Evidence-Based Policy, 2007). Second, higher data resolution helps identify specific intervention strengths and weaknesses. Strand analysis in this study revealed targeted student gains. Combination with multiple linear regression enabled Lincoln “within-cohort” growth analysis correlated with InSciEd Out. The inclusion of engagement metrics additionally extended didactic knowledge gains towards preliminary quantification of student entry into science. Engagement with the science pipeline is predictive of further STEM pipeline progression (Osborne, 2007; Aschbacher et *al.,* 2010).

Student data is foundational to improvement of the educational system despite research and practice having limited integration in the field of education (Porter and McMaken, 2009; Rust, 2009). Appropriate measures to assess science education practice is a topic of contention, but US students’ flagging performance on international achievement tests (Organisation for Economic Co-Operation and Development, 2012; Provasnik et *al.,* 2012) is an opportunity to iteratively improve our utility of US education resources. Better quantification of science education interventions is both possible and necessary for sustainable policymaking and continued betterment of student education.

## Supplementary Material

supp

## Figures and Tables

**Table 1 |:** Excerpt from the curriculum module rubric

Area of assessment	Expected outcome
STEM standards	Standards and benchmarks are identified and appropriate to the module; also lists standards from other subject- area disciplines integrated into the module. Next-Generation Standards are cooperatively identified and cross- mapped to current MN standards
Content objectives	Objectives are appropriate to the module and address recall and interpretation levels of knowledge (Bloom’s: Knowledge, Comprehension and Application)
Language objectives	Identifies the academic language and procedural discourse embedded within each lesson in the module and overtly incorporates a form of communication authentic to scientific community (that is, journal article, field notes, poster presentation, letter to legislator, webpage)
Horizontal integration	Curricular plan authentically integrates standards/objectives from other content areas in a manner that lends coherence
Health behaviour	An analysis of meaningful behavioural change is included in the module to capture health and behaviour outcomes of and beyond the students

Structural components	
5E lens of module	Clearly conveys 5E scope and sequence of the module
Standards lens of module	State and National Standards included in the module are clearly documented. Treatment of Next-Generation Standards is included to maintain National scope
Daily lesson lens	Clearly conveys the module from the lens of chronological lesson plans
Supporting documents (handouts,	Includes copies of all material (handouts, assessments, task cards) that will be distributed to students. Has detailed
task cards)	notes on how/when such materials will be used

*Note*: Different areas of InSciEd Out curricula and their expected outcomes are listed for key curricula foci. This gives an overview of key expectations of InSciEd Out curricula.

**Table 2 |:** Matched change in MCA Science broken down by strand

		*Cohort 2 (2009–2012)*	*Cohort 3 (2010–2013) Δ* z-score	*Cohort 4 (2011–2014)*
		
Overall	L	−0.253	−0.239	−0.016
	1	0.023	0.009	−0.028
	2	−0.082	−0.206	−0.118
	3	−0.074	−0.059	0.017
	4	−0.180	−0.054	0.086
	D	−0.096	−0.150	−0.032
HNS	L	−0.131	0.101	0.197
NSE	1	−0.050	−0.037	0.026
	2	−0.252	−0.154	−0.127
	3	0.040	−0.084	−0.094
	4	−0.164	−0.007	−0.020
	D	−0.138	−0.096	−0.048
PSCS	L	−0.232	−0.376	0.072
	1	−0.039	−0.097	−0.155
	2	−0.021	−0.298	−0.130
	3	0.112	0.016	0.164
	4	−0.257	−0.047	−0.233
	D	−0.091	−0.133	−0.016
ESS	L	−0.152	−0.423	−0.399
	1	−0.038	0.065	0.103
	2	0.139	−0.220	−0.102
	3	0.046	−0.055	0.085
	4	−0.126	−0.062	0.046
	D	−0.031	−0.103	0.010
LIFS	L	0.107	0.391	0.200
	1	0.103	−0.039	−0.263
	2	0.059	−0.028	−0.121
	3	0.301	0.032	−0.070
	4	0.043	0.112	0.254
	D	0.085	−0.003	−0.087

*Note*: Comparisons are provided between Lincoln (L), District middle schools (1–4) and District (D). Units are state-normalized *z*-scores, which represent number of standard deviations above or below the mean. Δ*z*-score is the difference between grade 8 *z*-score and grade 5 *z*-score, with positive Δ*z*-score indicating “within-cohort” gains. See supplementary Table S3 for raw *z*-scores.

**Table 3 |:** Lincoln enrolment predictor contribution to multiple regression models

		Cohort 2 (2009–2012)	Cohort 3 (2010–2013)	Cohort 4 (2011–2014)	Overall (all cohorts)
All strands	*R*^2^	0.631	0.641	0.661	0.643
	*β* (SE)	−0.040 (0.112)	−0.010 (0.117)	0.173 (0.093)	0.057 (0.061)
	Δ*R*^2^	0.000	0.000	0.001	0.000
	*P* of Δ*R*^2^	0.721	0.929	0.064	0.354
HNS	*R*^2^	0.430	0.428	0.460	0.437
NSE	*β* (SE)	0.150 (0.136)	0.217 (0.143)	0.375** (0.119)	0.266***(0.075)
	Δ*R*^2^	0.001	0.001	0.005	0.002
	*P* of Δ*R*^2^	0.268	0.129	0.002**	0.000***
LIFS	R^2^	0.298	0.386	0.367	0.343
	*β* (SE)	0.196 (0.152)	0.452*** (0.144)	0.471*** (0.131)	0.385*** (0.082)
	Δ*R*^2^	0.001	0.007	0.008	0.005
	*P* of Δ*R*^2^	0.198	0.002**	0.000***	0.000***
PSCS	*R*^2^	0.337	0.398	0.329	0.351
	*β* (SE)	0.059 (0.156)	0.069 (0.146)	0.253 (0.130)	0.143 (0.082)
	Δ*R*^2^	0.000	0.000	0.003	0.001
	*P* of Δ*R*^2^	0.705	0.637	0.051	0.081
ESS	*R*^2^	0.374	0.365	0.312	0.348
	*β* (SE)	0.055 (0.145)	−0.089 (0.151)	−0.008 (0.134)	−0.014 (0.082)
	Δ*R*^2^	0.000	0.000	0.000	0.000
	*P* of Δ*R*^2^	0.703	0.556	0.950	0.862

Significance:

*** *P*<0.001

** *P*<0.01

* *P*<0.05.

*Note: R*^2^ is model explained variance; *p* (SE) is mean (standard error) contribution of Lincoln enrolment, reported in *z*-scores; Δ*R*^2^ is explained variance attributable to Lincoln enrolment; *P*-values are from *F*-tests to quantify significance of Lincoln enrolment-attributable increase in explained variance. Other predictors include gender (female), English learner, special education, free or reduced priced lunch, ethnicity (Hispanic), race (Black) and previous strand *z*-score. See supplementary Table S4 for full models.
