# Supplementary material for: Students being and becoming scientists: measured success in a novel science education partnership
Source: Palgrave Commun. Author manuscript; Available in PMC 2019 Jun 7. (PMC6555486; doi:10.1057/palcomms.2016.5)
Supplement: supp [file NIHMS1023239-supplement-supp.pdf]

Supplementary Information for:

Science Education InSciEd Out: Intervening for Growth in Student Scientific Engagement and Learning

Joanna Yang[1]*, Thomas J. LaBounty[2]*, Stephen C. Ekker[1,3], Chris Pierret[1,3]

[1]Clinical and Translational Science, Mayo Clinic, Rochester, MN.

[2]LaBounty Consulting, LLC, Woodbury, MN

[3]Biochemistry and Molecular Biology, Mayo Clinic, Rochester, MN.

*These authors contributed equally to this work

	06-07	07-08	08-09	09-10	10-11	11-12	12-13	13-14	14-15
# Participants	10	4	8	41	60	88	91	116	118
Total # Eligible	83	86	97	99	106	107	114	122	127
% Participation	12%	5%	8%	41%	57%	82%	80%	95%	93%

Table S1. Raw Data for Science Fair Election. Raw data for numbers of participating Lincoln students and total eligible Lincoln students (number of students in grades 6 to 8 at Lincoln) is provided for each year of analysis. Percent participation is calculated as the proportion of participants out of those eligible in any given year.

	06-07	07-08	08-09	09-10	10-11	11-12	12-13	13-14	14-15
# Enrolled	10	11	9	23	33	27	26	40	35
Total # Eligible	27	27	30	27	38	32	31	41	37
% Enrollment	37%	41%	30%	85%	87%	84%	84%	98%	95%

Table S2. Raw Data for Honors Biology Enrollment. Raw data for numbers of enrolled Lincoln students and total eligible Lincoln students (number of students in grade 8 at Lincoln) is provided for each year of analysis. Percent enrollment is calculated as the proportion of enrollees out of those eligible in any given year.

	N	Overall 5	Overall 8	Overall Δ	HSN/NSE 5	HSN/NSE 8	HSN/NSE Δ	PSCS 5	PSCS 8	PSCS Δ	ESS 5	ESS 8	ESS Δ	LIFS 5	LIFS 8	LIFS Δ
**Cohort 2 (2009-2012)**																
**L**	32	0.664	0.411	-0.253	0.502	0.371	-0.131	0.655	0.423	-0.232	0.430	0.278	-0.152	0.372	0.479	0.107
1	273	0.156	0.133	-0.023	0.154	0.104	-0.050	0.216	0.177	-0.039	0.026	-0.012	-0.038	0.089	0.192	0.103
2	269	0.162	-0.018	-0.180	0.160	-0.004	-0.164	0.188	-0.069	-0.257	0.037	-0.089	-0.126	0.041	0.084	0.043
3	239	0.363	0.281	-0.082	0.377	0.125	-0.252	0.381	0.360	-0.021	0.144	0.283	0.139	0.156	0.215	0.059
4	125	0.491	0.565	0.074	0.370	0.410	0.040	0.455	0.568	0.112	0.342	0.387	0.046	0.210	0.511	0.301
D	968	0.246	0.151	-0.096	0.230	0.092	-0.138	0.272	0.181	-0.091	0.098	0.067	-0.031	0.097	0.182	0.085
**Cohort 3 (2010-2013)**																
**L**	31	0.877	0.638	-0.239	0.395	0.496	0.101	1.012	0.636	-0.376	0.790	0.368	-0.423	0.440	0.832	0.391
**1**	234	0.059	0.068	0.009	0.042	0.005	-0.037	0.101	0.005	-0.097	0.013	0.079	0.065	0.101	0.062	-0.039
**2**	270	0.129	-0.076	-0.206	0.034	-0.120	-0.154	0.258	-0.041	-0.298	0.093	-0.127	-0.220	0.079	0.050	-0.028
**3**	234	0.301	0.242	-0.059	0.237	0.153	-0.084	0.275	0.290	0.016	0.181	0.126	-0.055	0.215	0.248	0.032
**4**	136	0.664	0.600	-0.054	0.482	0.475	-0.007	0.496	0.449	-0.047	0.426	0.365	-0.062	0.448	0.560	0.112
**D**	932	0.282	0.132	-0.150	0.151	0.055	-0.096	0.260	0.127	-0.133	0.154	0.051	-0.103	0.179	0.176	-0.003
**Cohort 4 (2011-2014)**																
**L**	41	0.683	0.667	-0.016	0.476	0.672	0.197	0.455	0.527	0.072	0.713	0.314	-0.399	0.536	0.735	0.200
**1**	265	-0.094	-0.122	-0.028	-0.035	-0.010	0.026	0.088	-0.066	-0.155	-0.299	-0.196	0.103	0.037	-0.227	-0.263
2	279	0.046	-0.072	-0.118	0.030	-0.097	-0.127	0.171	0.040	-0.130	-0.142	-0.244	-0.102	0.137	0.016	-0.121
3	217	0.207	0.225	0.017	0.214	0.121	-0.094	0.162	0.326	0.164	0.080	0.164	0.085	0.205	0.135	-0.070
4	143	0.610	0.696	0.086	0.564	0.544	-0.020	0.447	0.680	0.233	0.402	0.448	0.046	0.476	0.730	0.254
D	977	0.134	0.101	-0.032	0.131	0.083	-0.048	0.185	0.169	-0.016	-0.037	-0.027	0.010	0.178	0.091	-0.087

Scores are state-centered z-scores in each year.

School percentiles based on all schools with at least 10 records in the state (N=816, N=509).

Table S3. Raw data for matched change in MCA Science broken down by strand.

Comparisons are provided between Lincoln (L), District middle schools (1-4), and District (D). Analysis utilized state-normalized z-scores, which represent number of standard deviations above or below the mean. Δ z-score is the difference between grade 8 z-score and grade 5 z-score, with positive Δz-score indicating “within-cohort” gains.

	Overall		HNS/NSE		LIFS		PSCS		ESS	
Predictor	β	SE	β	SE	β	SE	β	SE	β	SE
Intercept	0.106**	0.036	0.185***	0.042	0.326***	0.047	0.248***	0.050	0.239***	0.045
Female	-0.111**	0.040	-0.168***	0.049	-0.061	0.055	-0.072	0.057	-0.141**	0.052
ESL	0.048	0.073	-0.126	0.088	-0.023	0.099	-0.262**	0.099	-0.088	0.094
SPED	-0.121	0.070	-0.23**	0.084	-0.269**	0.096	-0.346***	0.096	-0.143	0.090
FRPL	-0.218***	0.054	-0.222***	0.065	-0.406***	0.071	-0.26***	0.073	-0.377***	0.068
Hispanic	-0.100	0.098	-0.121	0.118	-0.052	0.133	-0.211	0.135	-0.162	0.126
Black	-0.049	0.076	-0.255**	0.091	-0.159	0.103	-0.12	0.106	-0.114	0.098
Prev. SS	0.731***	0.023	0.52***	0.027	0.431***	0.032	0.48***	0.032	0.503***	0.031
Lincoln	-0.040	0.112	0.15	0.136	0.196	0.152	0.059	0.156	0.055	0.145
R^2	0.631		0.430		0.298		0.337		0.374	
ΔR^2	0.000		0.001		0.001		0.000		0.000	
F of ΔR^2	0.127		0.127		1.66		0.144		0.146	

	Overall		HNS/NSE		LIFS		PSCS		ESS	
Predictor	β	SE	β	SE	β	SE	β	SE	β	SE
Intercept	0.152***	0.039	0.211***	0.045	0.364***	0.045	0.274***	0.048	0.309***	0.047
Female	-0.164***	0.042	-0.106*	0.051	-0.12*	0.052	-0.216***	0.053	-0.217***	0.054
ESL	-0.070	0.071	-0.232**	0.087	-0.391***	0.087	-0.242**	0.088	-0.208*	0.091
SPED	-0.093	0.071	-0.136	0.087	-0.347***	0.085	-0.243**	0.087	-0.275**	0.089
FRPL	-0.217***	0.055	-0.312***	0.068	-0.31***	0.068	-0.239***	0.069	-0.372***	0.070
Hispanic	-0.021	0.086	-0.078	0.106	0.046	0.107	-0.027	0.108	-0.158	0.111
Black	-0.113	0.072	-0.239**	0.088	-0.227*	0.089	-0.178	0.091	-0.174	0.093
Prev. SS	0.694***	0.024	0.487***	0.030	0.413***	0.029	0.458***	0.030	0.424***	0.030
Lincoln	-0.01	0.117	0.217	0.143	0.452**	0.144	0.069	0.146	-0.089	0.151
R^2	0.641		0.428		0.386		0.398		0.365	
ΔR^2	0.000		0.001		0.007		0.000		0.000	
F of ΔR^2	0.008		2.308		***9.857****		0.222		0.347	

	Overall		HNS/NSE		LIFS		PSCS		ESS	
Predictor	β	SE	β	SE	β	SE	β	SE	β	SE
Intercept	0.142***	0.033	0.276***	0.041	0.183***	0.046	0.339***	0.047	0.182***	0.046
Female	-0.103**	0.037	-0.191***	0.048	-0.056	0.053	-0.157**	0.053	-0.134*	0.054
ESL	-0.121	0.068	-0.21*	0.086	-0.253**	0.095	-0.27**	0.094	-0.166	0.095
SPED	-0.152*	0.067	-0.215*	0.086	-0.216*	0.093	-0.407***	0.093	-0.202*	0.095
FRPL	-0.195***	0.050	-0.315***	0.063	-0.344***	0.069	-0.297***	0.068	-0.205**	0.070
Hispanic	0.107	0.087	-0.123	0.111	0.113	0.123	0.096	0.122	-0.156	0.124
Black	-0.033	0.066	-0.201*	0.085	-0.119	0.093	-0.119	0.093	-0.052	0.095
Prev. SS	0.717***	0.021	0.542***	0.027	0.493***	0.029	0.417***	0.030	0.44***	0.029
Lincoln	0.173	0.093	0.375**	0.119	0.471***	0.131	0.253	0.130	-0.008	0.134
R^2	0.661		0.460		0.367		0.329		0.312	
ΔR^2	0.001		0.005		0.008		0.003		0.000	
F of ΔR^2	3.430		**9.950****		**12.974*****		3.801		0.004	

	Overall		HNS/NSE		LIFS		PSCS		ESS	
Predictor	β	SE	β	SE	β	SE	β	SE	β	SE
Intercept	0.133***	0.021	0.221***	0.024	0.298***	0.027	0.29***	0.028	0.247***	0.026
Female	-0.125***	0.023	-0.153***	0.028	-0.083**	0.031	-0.147***	0.031	-0.165***	0.031
ESL	-0.051	0.041	-0.191***	0.050	-0.231***	0.054	-0.261***	0.054	-0.164**	0.054
SPED	-0.123**	0.040	-0.185***	0.049	-0.279***	0.053	-0.336***	0.053	-0.219***	0.053
FRPL	-0.207***	0.030	-0.282***	0.037	-0.362***	0.040	-0.262***	0.040	-0.316***	0.040
Hispanic	-0.003	0.052	-0.108	0.064	0.041	0.070	-0.039	0.069	-0.154*	0.069
Black	-0.067	0.041	-0.233***	0.051	-0.171**	0.055	-0.144**	0.055	-0.116*	0.055
Prev. SS	0.712***	0.013	0.513***	0.016	0.446***	0.017	0.444***	0.018	0.454***	0.017
Lincoln K	0.057	0.061	0.266***	0.075	0.385***	0.082	0.143	0.082	-0.014	0.082
R^2	0.643		0.437		0.343		0.351		0.348	
ΔR^2	0.000		0.002		0.005		0.001		0.000	
F of ΔR^2	0.860		**12.412*****		**21.9*****		3.047		0.03	

Table S4. Full multiple regression models for all versus individual strands across all cohorts. R^2 is model explained variance; β (SE) is the mean (standard error) contribution of each predictor, reported in z-scores; ΔR^2 is explained variance attributable to Lincoln enrollment; F of ΔR^2 quantifies significance of Lincoln enrollment-attributable increase in explained variance. ESL is English as a Second Language, SPED is Special Education, FRPL is Free and Reduced Price Lunch, and Prev. SS is Previous Strand Score.
